# Restricted sweat evaporation preceding short term heat acclimation accelerates adaption in females

**DOI:** 10.1186/2046-7648-4-S1-A112

**Published:** 2015-09-14

**Authors:** Jessica Mee, Sophie Peters, Jo Doust, Neil Maxwell

**Affiliations:** 1 Environmental Extremes Lab, Centre for Sport and Exercise Science and Medicine, University of Brighton, Eastbourne, UK

## Introduction

Short term heat acclimation (STHA) is a preferred regime for athletes, since it is easier to adopt when sustaining quality training and tapering performance in the weeks prior to competition. Females have been reported to establish an enhance sudomotor function following STHA; however, they require long term HA to establish cardiovascular and thermoregulatory adaptation [1]. The current study, assessed the effectiveness of five days of controlled hyperthermia HA, combined with a restricted sweat evaporation exposure, to elicit thermoregulatory, cardiovascular and sudomotor adaptation.

## Method

Nine females performed two running heat tolerance tests (RHTT) [2], separated by five controlled hyperthermia (Tr ~ 38.5°C) HA sessions. For 20 minutes before HA, participants were exposed to a temperate environment (HA) or a hot environment (50°C, 30% RH), whilst wearing a 100% Vinyl sauna suit (HA_sauna_). Conditions were performed in a balanced randomised order and separated by ~7 weeks. Testing was completed during the follicular phase of the menstrual cycle or the pill free phase of oral contraception use; confirmed by plasma concentrations of 17β-estradiol and progesterone. A two-way repeated measures ANOVA was performed to identify difference in the physiological characteristics during the RHTT, between the HA and HA_sauna _conditions. When a main effect or interaction effect was found, results were followed up using Bonferroni corrected post hoc comparisons.

## Results

In the HA_sauna _condition, resting rectal temperature (Tr_rest_) (-0.28(0.15) °C), resting heart rate (HR_rest_) (-9 (4) beats.min^-1^), peak rectal temperature (Tr_peak_) (-0.42(0.22) °C), peak heart rate (HR_peak_) (-12(7) beats.min^-1^) peak skin temperature (Tskin_peak_) (-0.89(0.86) °C), sweat ion concentration (-16(10) mmol.L^-1^) and sweat-onset Tr (-0.26(0.15) °C) reduced, sweat rate (SR) (+565(197) g.hr^-1^) and forearm SR (SR_forearm_) (+0.15(0.14) mg.cm^2^/min) (*p *≤ 0.05) increased. In the HA condition, HR_peak _(-4(5) beats.min^-1^) and sweat ion concentration (-8(4) mmol.L^-1^) reduced; and SR increased (+462(399) g.hr^-1^) (*p *≤ 0.05); while no differences were observed in Tr_rest_, Tr_peak_, HR_rest_, Tskin_peak_, SR_forearm _and sweat-onset Tr. Plasma volume expansion was greater following the HA_sauna _condition (9.3(7.6) % vs. 1.3(5.0) %; *p *≤ 0.05).

## Discussion

HA_sauna _was effective in attenuating thermoregulatory and cardiovascular strain; this was not achieved following the HA alone. Exercise was matched for metabolic heat production thus; the reduced thermoregulatory strain was potentially due to an increased evaporative heat loss, resulting in a reduction in heat storage as a result of altered afferent neural activity from the peripheral or central thermo-receptors. The reduced cardiovascular strain following HA_sauna _can be explained by the plasma volume expansion, suggesting an increased blood volume, thus preserving stroke volume and reducing heart rate at a given workload.

## Conclusions

This study suggests females should consider including a period of restricted sweat evaporation prior to HA sessions to promote an accelerated thermoregulatory, cardiovascular and sudomotor adaptation.
